# Effect of lunch with different calorie and nutrient balances on dinner-induced postprandial glucose variability

**DOI:** 10.1186/s12986-022-00704-1

**Published:** 2022-09-24

**Authors:** Mai Kuwahara, Hyeon-Ki Kim, Akiko Furutani, Yui Mineshita, Takashi Nakaoka, Shigenobu Shibata

**Affiliations:** 1 Graduate School of Advanced Science and Engineering, Waseda University, 3-4-1 Ohkubo, Shinjuku-ku, Tokyo, Japan; 2 Faculty of Science and Engineering, Waseda University, 2-2 Wakamatsu, Shinjuku-ku, Tokyo, Japan; 3 Japan Organization of Occupational Health and Safety, Kawasaki, Kanagawa, Japan

**Keywords:** Lunch, Energy intake, Energy balance, Starving time, Glucose level

## Abstract

**Aim:**

This study aimed to examine the effect of lunches with different caloric contents (Study 1) and nutrient balances (Study 2) on dinner-induced postprandial glucose fluctuation.

**Methods:**

Energy trial (Study 1): Thirteen healthy young participants (n = 10 men, n = 3 women) were investigated to determine the effects of different caloric intakes at lunch on glucose level variability. The study was comprised of four trials (no lunch, low lunch, standard lunch, and high-energy lunch). Energy balance trial (Study 2): Fourteen healthy young adults (n = 8 men, n = 6 women) were investigated to determine the effect of different nutrient balances during lunch on glucose level variability. The study consisted of four trials (standard, protein-rich, fat-rich, and carbohydrate-rich). In studies 1 and 2, each trial was spaced at least 24 full hours apart, and breakfast and dinner were tested as meals. The mealtimes for each trial were then aligned. Continuous glucose monitoring was used to assess the blood glucose fluctuations.

**Results:**

Study 1: The no-lunch (95% CI 95.5–149.7) and low-energy lunch (95% CI 90.8–143.1) trials had significantly higher values in the incremental area under the curve (iAUC) of postprandial blood glucose at dinner compared to the standard (95% CI 55.4–90.0) and high-energy lunch (95% CI 29.3–54.6) trials (*P* = 0.006, *P* = 0.001 vs. none), (*P* = 0.004, *P* = 0.001 vs. low-energy trial). Study 2: A significantly higher postprandial blood glucose iAUC for dinner was found in the fat-rich trial (95% CI 58.5–114.0) than that in the protein-rich (95% CI 25.6–63.9) and standard (95% CI 25.6–112.4) trials, (*P* = 0.006, *P* = 0.035 vs. fat-rich trial).

**Conclusions:**

Our findings indicate that skipping lunch and low-calorie or high-lipid intake increased postprandial blood glucose levels after dinner.

**Supplementary Information:**

The online version contains supplementary material available at 10.1186/s12986-022-00704-1.

## Introduction

Previous studies on meal patterns in people with obesity have focused primarily on the association between meal size [1], timing [2], and frequency [3]. Recently, in a systematic review and network meta-analysis that examined the relationship between meal frequency and obesity [3], one meal per day was ranked as the best frequency for reducing body weight, followed by two meals per day, but not by three meals per day, compared with > 8 meals per day. In contrast, two meals per day performed best for the reduction of waist size compared with six meals per day [3]. This review suggests that 3 meals per day may not be the best way to control body weight. Several studies have suggested that habitual breakfast skipping is related to health problems such as the risk of obesity [4], cardiovascular diseases [5], and cognitive function [6]. A survey of feeding patterns in Japan reported that many people have a meal ratio (10 for whole day’s energy intake) of 2.5 for breakfast; 3, lunch; and, 4.5, dinner [7]. At dinner, obesity is positively related to not only the energy quantity of meals but also the eating time of meals. These studies suggest a relatively large energy quantity of breakfast and a relatively small energy quantity of dinner for health promotion. However, few reports have demonstrated the role of lunch in health promotion.

Little is known about the relationship between employees’ meal-skipping patterns and workplace dietary choices and health [8]. Employees who skipped breakfast on > 3 days/week had lower healthy purchase index (HPI) compared with those who never skipped breakfast [8]. In addition, skipping lunch on > 3 days/week and dinner > 1 day/week were associated with significantly lower HPC than never skipping lunch. This study suggests that not skipping lunch may help with the purchase of a healthier meal for lunch. During the COVID-19 pandemic lockdowns, practices of healthy eating, skipping lunch, and more frequent physical activity were significantly associated with weight loss [9]. Although breakfast skipping is related to obesity, this study suggests the possibility of skipping lunch for weight loss. However, there are no detailed reports on the effect of lunch skipping and/or unhealthy lunch on postprandial glucose levels at dinner. In Iran in 2015, a cross-sectional nationwide study (14,286 students aged 7–18) revealed that the frequencies of breakfast, lunch, and dinner skipping were 13.85, 6.8%, and 7.5%, respectively [10]. Thus, the percentage of lunch skipping is not near 0%, but approximately half as that of breakfast skipping, and, lunch skipping should be taken into consideration in addition to breakfast skipping.

Several studies have demonstrated that water-soluble dietary fiber consumption [11–13], low-glycemic index meal consumption [14, 15], and protein-rich meal consumption [16, 17] can influence the postprandial glycemic response at the next meal. This effect has led to the glucose levels following the second meal being attenuated. When fiber-rich food is consumed at breakfast as a first meal, the following second meal, such as lunch, it attenuates the increase in glucose levels compared with the usual food [18]. In a recent cross-over experiment in humans, we demonstrated that the consumption of 5 g dry powder (approximately 50% inulin) of artichokes at breakfast time attenuated glucose increase after lunch and dinner compared with no artichoke intake [19]. Furthermore, snacks after lunch have also been reported to affect glucose level changes at dinner time. Fiber-rich snacks attenuate tissue glucose increase at dinner time in healthy individuals [20, 21]. Moreover, consuming snacks in the late afternoon attenuated the tissue glucose level at dinner in patients with type 2 diabetes [22]. Thus, lunch may affect the glucose level change at dinner as a second-meal effect.

Several previous studies have revealed that the increase in blood glucose is higher during lunch and/or dinner than during breakfast [23, 24]. In addition, in our recent crossover trial in which participants ate the same meals at breakfast or dinner times on different days, the postprandial tissue glucose increase was higher at the dinner meal than at the breakfast meal, suggesting circadian clock control of glucose level through insulin release and/or insulin receptor sensitivity [25]. Tachyphagia (a meal lasting < 15 min) rapidly increases blood glucose levels and promotes weight gain. Recently, it was reported that eating while standing and eating fast food at lunchtime were positively associated with tachyphagia [26]. Previous studies have reported that meal skipping can affect postprandial blood glucose levels in the next meal [27–30]. Thus, the meal composition of lunch may affect the postprandial glucose increase through tachyphagia.

We aimed to investigate the amount of energy and components of lunch for controlling glucose levels at dinner. Therefore, in the first part of the current study, we examined whether the amount of energy change from 0 kcal to approximately 1000 kcal at lunch as the first meal affected postprandial glucose levels after dinner as the second meal. Second, we examined whether the standard amount of energy of lunch with unbalanced nutrients (protein-rich, fat-rich, or carbohydrate-rich) affected postprandial glucose levels after dinner.

## Materials and methods

### Participant

This study included healthy young adults (*N* = 32; 21 men and 11 women) from Tokyo (Japan). The study was conducted between April 2021 and February 2022, and the inclusion criteria were as follows: (1) not using glucose/insulin-lowering or related medications; (2) not having doctor-diagnosed diabetes; and (3) not taking antidiabetic supplements. This study was conducted in accordance with the Helsinki Declaration and was approved by the Ethics Committee for Humans at Waseda University (approval number: 2020-371). The human trial of the current study is registered at UMIN (www.umin.ac.jp/ctr/ number: UMIN000043287). Informed consent was obtained from all the participants after the experiment was described in detail.

**Fig. 1:**
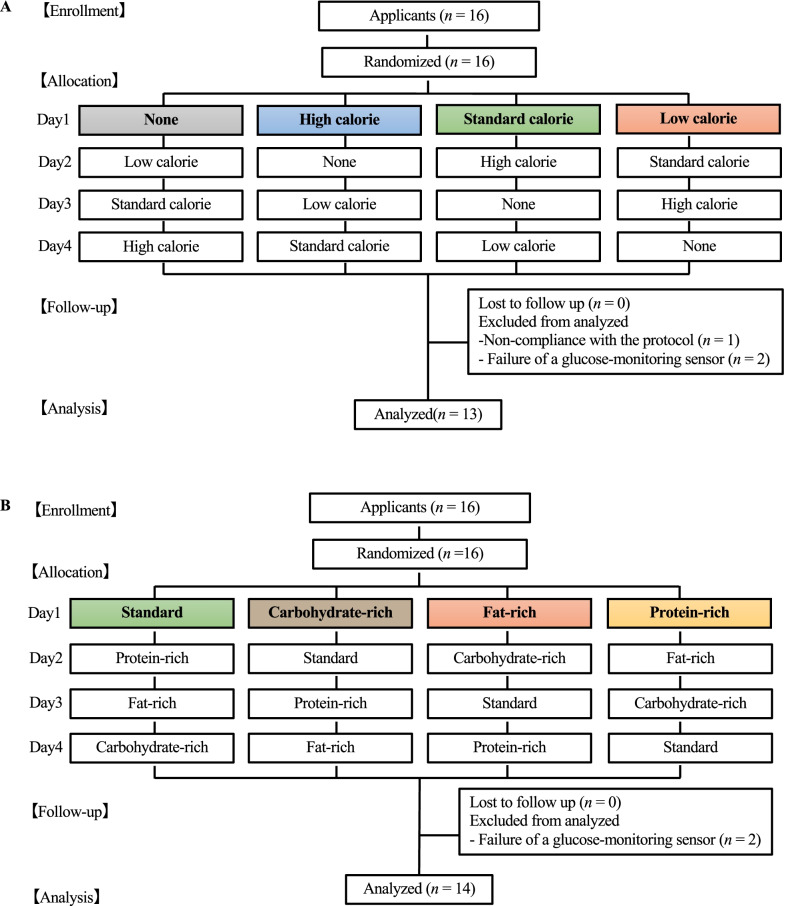
Consolidated standards of reporting trials flow diagram. The energy trial group (**A**) and the balance trial group (**B**)

Participants were excluded from the study for the following reasons: non-compliance with the protocol (*n* = 1) and failure of a glucose-monitoring sensor (*n* = 4). Consequently, twenty-seven participants were included in the final analysis (Fig. 1).

### Study design

A randomized crossover design was used in this study, with each participant consuming four different lunches. The participants were randomly assigned to the following two groups: the energy trial group (*n* = 13, *n* = 10 men and *n* = 3 women; Table 1A) and the balance trial group (*n* = 14, *n* = 8 men and *n* = 6 women; Table 1B). For the energy trial, the protein, fat, and carbohydrate (PFC) balance at lunch was similar, but blood glucose fluctuations were examined when caloric intake was changed. Four types of lunches were prepared in the energy trial: (1) no, (2) low, (3) standard, and (4) high-energy lunch trials. On the other hand, for the balance trial, the caloric intakes at lunch were similar; however, the PFC balance was changed and subsequent blood glucose level fluctuations were examined. Four types of lunches were prepared in the nutritional balance trial: (1) standard, (2) protein-rich, (3) fat-rich, and (4) carbohydrate-rich.

**Table 1:** Characteristics of participants

	A		B	
	Men (*n* = 10)	Women (*n* = 3)	Men (*n* = 8)	Women (*n* = 6)
Age (years)	22.4(4.0)	21.7(0.5)	24.6(5.6)	23.8(4.6)
Height (cm)	174.6(5.9)	160.7(4.2)	172.5(4.5)	159.8(6.5)
Body weight (kg)	72.0(10.5)	54.9(6.4)	59.7(11.4)	53.7(5.3)
BMI (kg/m^2^)	23.7(1.1)	21.3(2.5)	19.9(2.9)	21.0(1.6)
Percentage fat (%)	21.2(7.6)	21.2(1.2)	21.5(7.7)	26.0(4.6)
Muscle mass (kg)	25.7(6.9)	15.9(4.9)	19.3(9.3)	19.1(1.6)
Body fat mass (kg)	14.4(5.7)	28.3(6.3)	13.5(5.3)	19.4(8.1)

All data are presented as the mean (standard deviation), BMI: body mass index, (A) Characteristics of participants for the energy trial group, (B) Characteristics of participants for the balance trial group

The trial for each group was structured into two weeks, with each trial spaced at least 24 full hours apart. As one of the purposes of the study was to examine blood glucose fluctuations in daily life, each participant was asked to follow his or her usual meal time and was instructed to align the times of breakfast, lunch, and dinner intake in each trial. Only designated meals were allowed in each trial. The diet was prepared based on the results of the National Health and Nutrition Survey in Japan [31]. Strenuous exercise was prohibited during the study period.

### Test meals

#### Test meals for breakfast and dinner

All trial breakfasts and dinners were provided as test meals. Fruit granola 60 g (Calbee, Inc., Tokyo, Japan) and Milk 200 mL (Morinaga & Co., Ltd., Tokyo, Japan) were served for breakfast. The energy intake was 399.8 kcal (Table 2A). For dinner, a Sukiyaki bento (Tokatsu Foods Co., Ltd., Kanagawa, Japan), Rice 200 g (Hagoromo Foods Co., Shizuoka, Japan), Hot spring egg 52 g (Lawson, Inc., Tokyo, Japan), and Miso soup (Marukome Co., Ltd., Nagano, Japan) were used. Miso soup was consumed by dissolving 18 g miso in 160 mL hot water for men or 36 g miso in 320 mL for women. The energy intake differed between men and women; it was 913.3 kcal for men and 655.5 kcal for women (Table 2B, C).

**Table 2:** Test meals for each trial

	A	B	C
Energy (kcal)	399.8	913.3	655.5
Protein (g)	11.7	36.0	25.7
(%)	11.4	15.8	15.7
Fat (g)	16.8	23.1	15.7
(%)	36.8	22.8	21.6
Carbohydrates (g)	53.1	140.1	102.3
(%)	51.7	61.5	62.6
-Dietary fiber (g)	5.4	4.3	4.6

	D				E			
Trial	None	Low	Standard	High	None	Low	Standard	High
Energy (kcal)	–	197.5	691.3	1185.0	–	158.0	526.0	895.3
Protein (g)	–	9.3	32.4	55.5	–	7.4	24.6	44.4
(%)		18.2	18.2	18.2		18.2	18.2	18.2
Fat (g)	–	8.3	28.9	49.5	–	6.6	22.0	39.6
(%)		36.4	36.4	36.4		36.4	36.4	36.4
Carbohydrates (g)	–	23.2	81.0	138.9	–	18.5	61.7	111.1
(%)		45.4	45.4	45.4		45.4	45.4	45.4
-Dietary fiber (g)	–	3.0	10.5	18.0	–	2.4	8.0 g	14.4

	F				G			
Trial	Standard	Protein-rich	Fat-rich	Carbohydrates-rich	Standard	Protein-rich	Fat-rich	Carbohydrates-rich
Energy (kcal)	691.3	694.0	694.8	716.2	526.0	503.0	514.8	540.2
Protein (g)	32.4	98.8	30.2	22.5	24.6	71.4	19.3	16.0
(%)	18.2	56.8	17.3	12.4	18.2	56.7	14.9	11.8
Fat (g)	28.9	21.2	52.4	10.1	22.0	15.5	37.4	7.2
(%)	36.4	27.4	67.7	12.5	36.4	27.7	65.2	11.9
Carbohydrates (g)	81.0	27.3	26.2	136.3	61.7	19.7	25.7	103.4
(%)	45.4	15.7	15.0	75.1	45.4	15.7	19.9	76.3
-Dietary fiber (g)	10.5	0.9	1.0	5.7	8.0	0.4	1.0	4.3

(A) Breakfast; Dietary contents of both men and women. The energy and the balance trials had the same meal contents for each trial. (B) Dinner contents for men; The energy and the balance trials had the same diet. (C) Dinner contents for women; The energy and the balance trials had the same diet. (D, E) Lunch contents of the energy trial for men (D) and women (E). (F, G) Lunch contents of the balance trial for men (F) and women (G). The contents of the standard trial were the same as those of the calorie trial. Fiber contents were included in the carbohydrate content in all of the trials.

#### Study meals for energy trial

The nutritional balance of lunch for each trial was set at P: 18.2%, F: 36.4%, and C: 45.4%. For the low-energy lunch trial, Calorie Mate Jelly 108 g for men or 86 g for women (Otsuka Pharmaceutical Co., Ltd., Tokyo, Japan) and Protein Bar Wafer Vanilla 19 g (Morinaga & Co., Ltd., Tokyo, Japan) for men or 15 g for women were served for lunch. For the standard-energy lunch trial, Calorie Mate Jelly 376 g for men or 286 g for women and Protein Bar Wafer Vanilla 65 g for men or 49 g for women were served for lunch. For the high-energy lunch trial, Calorie Mate Jelly 645 g for men or 486 g for women and Protein Bar Wafer Vanilla 111 g for men or 84 g for women were served for lunch. Details of the meals for each trial are shown in Table 2D, E.

#### Study meals for balance trial

For the standard trial, Calorie mate jelly 376 g for men or 286 g for women (Otsuka Pharmaceutical Co., Ltd., Tokyo, Japan) and a Protein bar 65 g for men or 49 g for women (Morinaga & Co., Ltd., Tokyo, Japan) were served for lunch. In the protein-rich trial, chicken salad 3 pieces for men 2 pieces for women (Lawson, Inc., Tokyo, Japan), Savas whey protein powder 21 g (Meiji Co., Ltd., Tokyo, Japan), Milk 200 mL (Morinaga & Co., Ltd., Tokyo, Japan), and Calorie mate bars 20 g for men or 6 g for women (Otsuka Pharmaceutical Co., Ltd., Tokyo, Japan) were served for lunch. In the fat-rich trial, Cheese 90 g for men or 36 g for women (Life Co., Tokyo, Japan), Peanuts 30 g (Life Co., Tokyo), and Calorie Mate bars 40 g (Otsuka Pharmaceutical Co., Ltd., Tokyo, Japan) were served for lunch. In the carbohydrate-rich trial, Papatto rice 200 g, (Hagoromo Foods Co., Shizuoka, Japan), Dried seasoning powder 5 g (Marumiya Foods Industries Co., Ltd., Tokyo, Japan), and Calorie mate Jelly 430 g for men or 280 g for women (Otsuka Pharmaceutical Co., Ltd., Tokyo, Japan) were served for lunch. The caloric intake and energy balance of the test meal varied between the male and female participants. The details of the meals for each trial are shown in Tables 2F and G.

### Measurements

#### Anthropometry and chronotype

Anthropometric variables were measured before the study. Height was measured to the nearest 0.1 cm with a portable Seca 213 height meter (As One Corporation, Osaka, Japan). Body weight was measured to the nearest 0.1 kg using InBody 270 (InBody Japan Corporation, Tokyo, Japan). Body mass index (BMI) was also calculated using InBody. Muscle mass, fat mass, and body fat percentage were measured by the bioelectrical impedance analysis (BIA) method using InBody 270 (InBody Japan Corporation, Tokyo, Japan).

The chronotype was assessed using the Horne–Ostberg Morningness–Eveningness Questionnaire (MEQ) [31], which consists of 19 questions related to preferred sleep time and daily performance. The sum yielded scores ranging from 16 to 86. Based on their scores, the participants were divided into three chronotype groups: morningness (score 59–86), intermediate (score 42–58), and eveningness (score 16–41).

#### Dietary intake

The participants’ energy intakes were assessed using a food frequency questionnaire (FFQ) [32]. The FFQ estimated the actual food intake by surveying the frequency and quantity of food intake using a questionnaire. The frequency and intake of breakfast, lunch, and dinner were surveyed, and the energy and nutrient intakes of the usual meals were calculated.

#### Physical activity levels

All the participants were asked to wear a triaxial accelerometer (Active style Pro HJA-750C; Omron Corp., Kyoto, Japan) during the study period. The participants were instructed to wear the activity meter at all times, except while bathing and sleeping. The data were valid only if the accelerometer was worn for at least 10 h (600 min) per day. Step count and moderate-to-vigorous physical activity (MVPA) were used for the assessment.

#### Glucose levels

On the first day of the study, a continuous glucose-monitoring (CGM) device, FreeStyle Libre Pro (Abbott Japan LLC, Tokyo, Japan), was placed on the participant's upper arm. After the study, the device was retrieved, and the data were read by a dedicated device. This device can measure glucose levels in the interstitial fluid every 15 min for 14 consecutive days. An observation period of at least half a day was established to stabilize glucose monitoring. For all trials, glycemic variability and peak glucose levels were calculated at a maximum of 4 h after lunch and dinner intake. The area under the curve (AUC) and incremental area under the curve (iAUC) were calculated from lunch and dinner consumption to 120, 180, and 240 min.

### Statistical analysis

All the data are presented as the mean (standard deviation). First, the Kolmogorov–Smirnov test was used to test for normality before statistical processing. Correlation analysis using the Pearson product-moment correlation coefficient was used to examine the relationship between the amount of physical activity and the standard trial for both the trials. One-way repeated-measures ANOVA was used to compare the AUC and iAUC after lunch and dinner for each trial. The Friedman test was used when normality was not observed. Correlation analysis with Pearson product-moment correlation coefficient was used to examine the relationship between the usual lunch intake or percentage of energy intake and after-dinner blood glucose levels in each trial. The Spearman test was used to examine the relationship between the differences in meal intake times for each trial in the energy trial and the after-dinner iAUC, as normality was not observed. On the other hand, Pearson’s product-moment correlation coefficient was used to examine the relationship between the difference in meal intake times and after-dinner iAUC for each trial in the balance trial, because normality was observed. A one-way ANOVA was also used to test the peak postprandial blood glucose values for each trial. The Friedman test was used when normality was not observed. A t-test was used to test comparisons of characteristics by mean starvation time. In addition, a two-way ANOVA was used to compare postprandial blood glucose variability between trials. All the data were analyzed using IBM SPSS Statistics statistical analysis software for Windows (SPSS Japan Inc., Tokyo, Japan). Statistical significance was set at *P* < 0.05.

## Results

### Relationship between the physical activity levels and AUC

In the energy trial, the step counts and MVPA per day during the study period were 9545.6 (4433.5) steps (95% CI 6603.4–1248.7) and 91.5 (39.1) min (95% CI 65.6–117.5). In the balance trial, the step counts and MVPA per day during the study period were 9905.8 (3546.6) steps (95% CI 7552.2–28.0) and 100.6 (28.1) min (95% CI 80.9–26.6–120.3) (Additional file 3: Fig. S3E, F). The correlation between physical activity level and the AUC of postprandial glucose level after dinner was analyzed during the study period. Step counts and MVPA were used as indices of physical activity level. In the energy trial, there was no significant correlation between step counts or MVPA during the study period and the after-dinner AUC in the standard lunch trial (Additional file 1: Fig. S1A, B). In the balance trial, there was no significant correlation between step counts or MVPA during the study period and the after-dinner AUC in the standard trial (Additional file 1: Fig. S1C, D).

### Comparison of the glucose levels in the energy trial

**Fig. 2:**
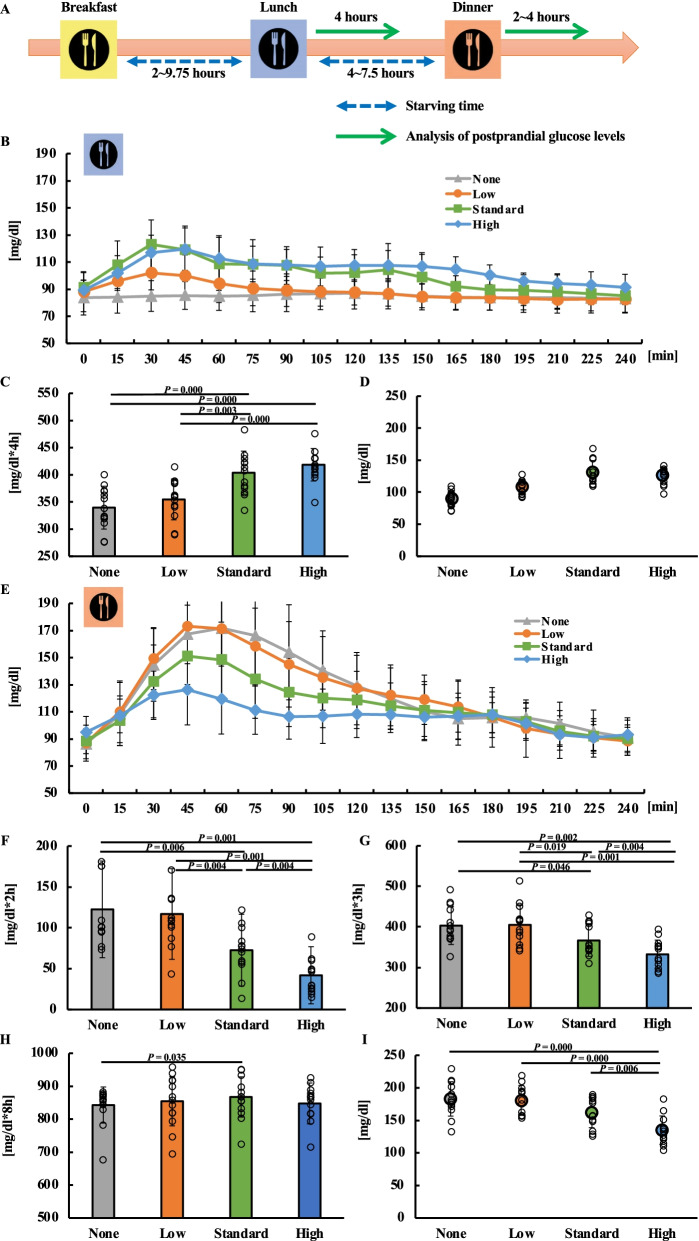
Postprandial blood glucose levels for lunch and dinner in the energy trial. All data are presented as the mean (standard deviation). Study protocol for the energy trial (**A**). Glucose concentration (**B**), area under the curve (AUC) (**C**), and peak blood glucose levels (**D**) for 4-h after lunch. Glucose concentration (**E**) for 4 h after dinner. Incremental AUC (iAUC) (**F**) and AUC (**G**) at 2 and 3 h after dinner. Sum of the AUC for 4 h after lunch and dinner for each trial in the energy trial (**H**). Peak blood glucose levels at 4 h after dinner (**I**). Figure 2C and I used One-way ANOVA. **D**, **F**, **G**, and **H** used the Friedman test.

The 24-h blood glucose excursion in the energy trial is shown in Additional file 2: Fig. S2A. The monitoring of glucose levels for 4 h after lunch showed an increase in postprandial blood glucose levels in the standard- and high-energy lunch trials (Fig. 2B). Significant differences between energy trials after lunch at each 15-min time interval were examined (Additional file 2: Supplemental Fig. S2C). The AUC for 4 h after lunch was 339.6 (39.4) (95% CI 315.8–363.4) in the no-energy lunch trial, 354.7 (37.5) (95% CI 332.0–377.3) in the low-energy lunch trial, 403.7 (39.7) (95% CI 379.7–427.7) in the standard-energy lunch trial and 418.4 (29.9) (95% CI 400.3–436.5) in high-energy lunch trial. The AUC for 4 h after lunch showed no significant difference between the no and low-energy lunch trials (*P* = 0.107) and between the standard- and high-energy lunch trials (*P* = 0.601, 95%CI = 16.2) (Fig. 2C). The peak for 4 h after lunch was 89.9 (12.1) (95% CI 82.6–97.3) in the no-energy lunch trial, 108.5 (11.0) (95% CI 101.9–115.2) in the low-energy lunch trial, 131.0 (18.3) (95% CI 119.9–142.1) in the standard-energy lunch trial and 126.5 (12.6) (95% CI 118.9–134.1) in high-energy lunch trial. The peak for 4 h after lunch showed a significant decrease in no-energy lunch trial compared with the low-, standard- and high-energy lunch trial (*P* = 0.004, *P* = 0.001, *P* = 0.001 vs no-energy lunch trial). The peak for 4 h after lunch showed a significant decrease in the low-energy lunch trial compared with the standard- and high-energy lunch trial (*P* = 0.001, *P* = 0.002 vs low-energy lunch trial). (Fig. 2D). Monitoring of glucose levels for 4 h after dinner showed an increase in postprandial blood glucose levels in the no- and low-energy lunch trials (Fig. 2E). Significant differences between energy trials after dinner at each 15-min time interval were examined (Additional file 2: Fig. S2D). The iAUC for 2 h after dinner was 122.6 (59.1) (95% CI 95.5–149.7) in the no-energy lunch trial, 117.0 (55.4) (95% CI 90.8–143.1) in the low-energy lunch trial, 72.7 (44.0) (95% CI 55.4–90.0) in the standard-energy lunch trial and 42.0 (34.9) (95% CI 29.3–54.6) in high-energy lunch trial. On examining the iAUC for 2 h after dinner, the no and low-energy lunch trials showed an increase in iAUC after dinner compared with the standard- and high-energy lunch trials (*P* = 0.006, *P* = 0.001 vs none), (*P* = 0.004, *P* = 0.001 vs low-energy trial) (Fig. 2F). The AUC for 3 h after dinner was 403.2 (46.5) (95% CI 375.1–431.3) in the no-energy lunch trial, 405.2 (49.2) (95% CI 375.4–434.9) in the low-energy lunch trial, 366.5 (39.0) (95% CI 342.9–390.0) in the standard-energy lunch trial and 332.4 (35.2) (95% CI 311.2–353.7) in high-energy lunch trial. On examining the AUC for 3 h after dinner, the standard-energy lunch trial had a significantly lower value than the no- and low-energy lunch trials (*P* = 0.046, *P* = 0.019 vs standard-energy lunch trial), and the high-energy lunch trial had a significantly lower value than the no-, low-, and standard-energy lunch trials (*P* = 0.002, *P* = 0.001 vs high-energy lunch trial) (Fig. 2G). The sum of the AUC for 4 h after lunch and dinner was 843.0 (54.8) (95% CI 808.5–877.4) in the no-energy lunch trial, 854.5 (74.7) (95% CI 807.5–901.5) in the low-energy lunch trial, 867.5 (61.1) (95% CI 829.1–906.0) in the standard-energy lunch trial and 847.8 (61.9) (95% CI 808.9–886.8) in high-energy lunch trial. On comparing the sum of the AUC for 4 h after lunch and dinner, the standard-energy lunch trial had a significantly higher value than the no-lunch trial (Fig. 2H). The peak glucose levels after dinner were 183.1 (26.4) (95% CI 167.1–199.0) in the no-energy lunch trial, 181.5 (22.1) (95% CI 168.1–194.8) in the low-energy lunch trial, 162.2 (23.3) (95% CI 148.1–176.2) in the standard-energy lunch trial, and 134.9 (21.9) (95% CI 127.2–148.1) in the high-energy lunch trial. The peak glucose levels after dinner in the no-, low-, and standard-energy lunch trials were significantly higher than that in the high-energy lunch trial (*P* = 0.000, *P* = 0.000, *P* = 0.006, vs high-energy lunch trial) (Fig. 2I).

### Comparison of the glucose levels in the balance trial

**Fig. 3:**
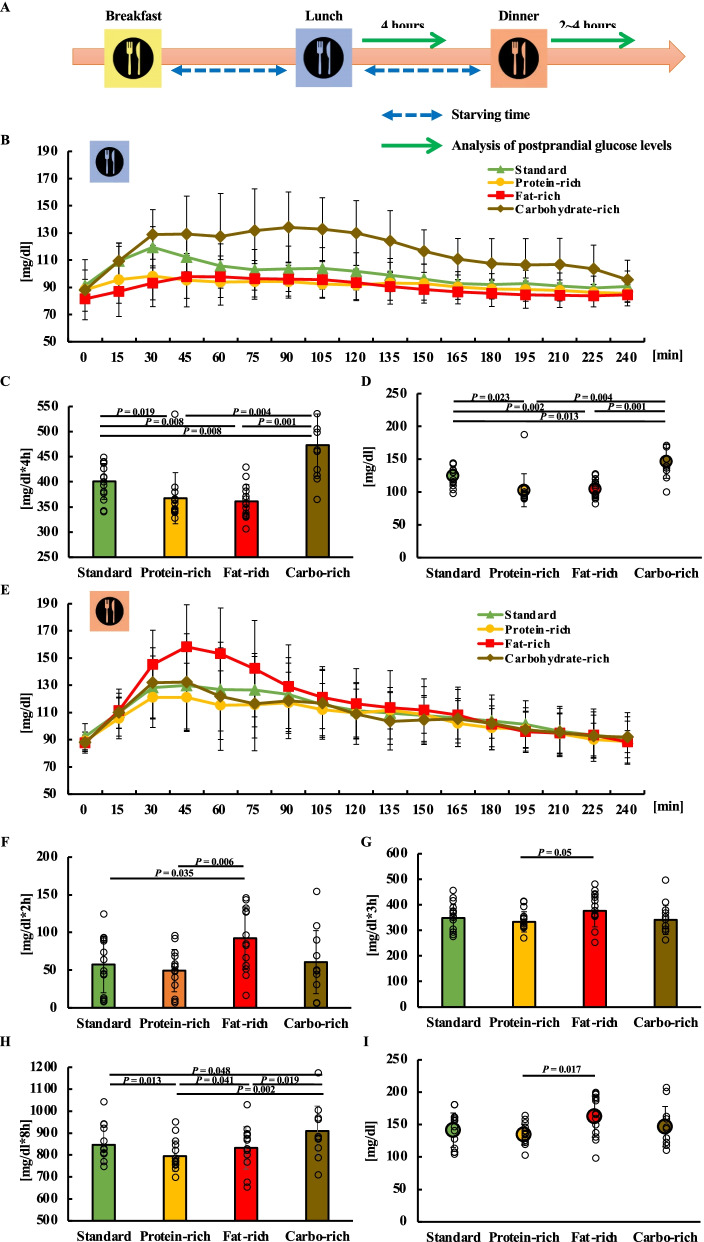
Postprandial blood glucose levels for lunch and dinner in the balance trial. All data are presented as the mean (standard deviation). Study protocol for the balance trial (**A**). Glucose concentration (**B**), area under the curve (AUC) (**C**), and peak blood glucose levels (**D**) for 4 h after lunch. Glucose concentration (**E**) for 4 h after dinner. Incremental AUC (iAUC) (**F**) and AUC (**G**) for 2 and 3 h after dinner. The sum of AUC for 4 h after lunch and dinner for each trial in the balance trial (**H**). Peak blood glucose levels at 4 h after dinner (**I**). Figure 3C, D, and H used the Friedman test. **F**, **G**, and **I** used One-way ANOVA.

The 24-h blood glucose excursion in the balance trial is shown in Additional file 2: Fig. S2B. In the balance trial, monitoring glucose levels for 4 h after lunch showed an increase in postprandial blood glucose levels in the carbohydrate-rich trial (Fig. 3B). Significant differences between balance trials after lunch at each 15-min time interval were examined (Additional file 2: Fig. S2E). The AUC for 4 h after lunch was 400.9 (35.6) (95% CI 380.3–421.5) in the standard trial, 367.5 (50.8) (95% CI 338.1–396.8) in the protein-rich trial, 361.2 (33.4) (95% CI 341.9–380.4) in the fat-rich trial and 473.0 (61.9) (95% CI 437.2–508.7) in the carbohydrate-rich trial. The AUC for 4 h after lunch showed a significant difference between the standard and other trials and between the carbohydrate-rich and other trials (*P* = 0.019 vs protein-rich trial, *P* = 0.006 vs fat-rich trial) (*P* = 0.006 vs standard trial, *P* = 0.004 vs protein-rich trial, *P* = 0.001 vs fat-rich trial) (Fig. 3C). The peak for 4 h after lunch was 124.9 (14.9) (95% CI 116.3–133.6) in the standard trial, 102.6 (25.1) (95% CI 88.1–117.1) in the protein-rich trial, 104.7 (14.3) (95% CI 96.5–113.0) in the fat-rich trial, and 146.7 (25.9) (95% CI 131.8–161.7) in the carbohydrate-rich trial. The peak for 4 h after lunch showed a significant increase in the carbohydrate-rich trial compared with the standard, protein-rich and fat-rich trial (*P* = 0.013, *P* = 0.004, *P* = 0.001 vs carbohydrate-rich trial). The peak for 4 h after lunch showed a significant increase in the standard trial compared with the protein-rich and fat-rich trial (*P* = 0.023, *P* = 0.002 vs standard trial) (Fig. 3D). Monitoring glucose levels for 4 h after dinner showed an increase in postprandial blood glucose levels in the fat-rich trial (Fig. 3E). Significant differences between balance trials after dinner at each 15-min time interval were examined (Additional file 2: Fig. S2F). The iAUC for 2 h after dinner was 57.5 (37.4) (95% CI 25.6–112.4) in the standard trial, 49.4 (28.0) (95% CI 25.6–63.9) in the protein-rich trial, 92.4 (41.4) (95% CI 58.5–114.0) in the fat-rich trial and 60.7 (41.8) (95% CI 29.1–84.9) in the carbohydrate-rich trial. On examining the iAUC for 2 h after dinner, there was a significantly higher rate in the fat-rich trials than those in the standard and protein-rich trials (*P* = 0.034, *P* = 0.022 vs fat-rich trials) (Fig. 3F). The AUC for 3 h after dinner was 348.4 (54.2) (95% CI 317.1–379.7) in the standard trial, 333.4 (39.8) (95% CI 310.4–356.4) in the protein-rich trial, 376.6 (62.6) (95% CI 340.4–412.7) in the fat-rich trial and 341.3 (57.3) (95% CI 308.2–374.4) in the carbohydrate-rich trial. A significantly higher AUC for 3 h after dinner was observed in the fat-rich trial than that in the protein-rich trial (*P* = 0.050 vs protein-rich trial) (Fig. 3G). The sum of the AUC for 4 h after lunch and dinner was 846.5 (80.9) (95% CI 799.8–893.2) in the standard trial, 794.8 (69.4) (95% CI 754.8–834.9) in the protein-rich trial, 832.4 (96.4) (95% CI 776.7–888.1) in the fat-rich trial, and 910.0 (113.2) (95% CI 844.4–975.2) in the carbohydrate-rich trial. On comparing the sum of the AUC for 4 h after lunch and dinner, the carbohydrate-rich trial had significantly higher values than the standard, protein-rich, and fat-rich trials. (*P* = 0.048, *P* = 0.002, *P* = 0.019 vs carbohydrate-rich trial) The number of protein-rich trials was significantly lower than that of the standard and fat-rich trials (*P* = 0.013, *P* = 0.041 vs protein-rich trial) (Fig. 3H). The peak blood glucose level after dinner was 141.4 (26.6) (95% CI 126.1–156.8) in the standard trial, 134.6 (16.0) (95% CI 125.3–143.8) in the protein-rich trial, 162.4 (31.8) (95% CI 144.1–180.8) in the fat-rich trial, and 146.9 (31.1) (95% CI 128.9–164.9) in the carbohydrate-rich trial The peak blood glucose level after dinner was significantly higher in the fat-rich trial than that in the protein-rich trial (*P* = 0.017 vs protein-rich trial) (Fig. 3I).

### Relationship between starvation time and blood glucose levels

#### Correlation between the difference in starvation time from breakfast to lunch and blood glucose levels

**Fig. 4:**
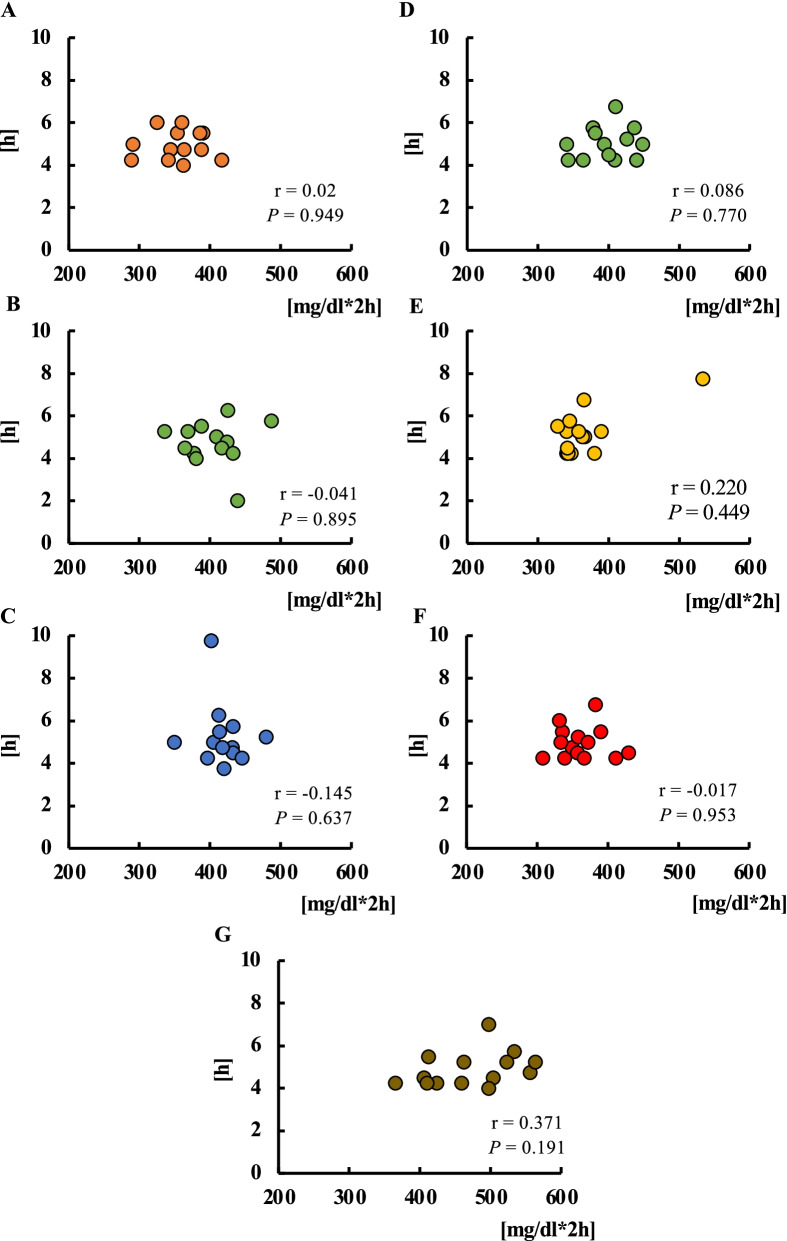
Correlations between starvation time from breakfast to lunch and postprandial blood glucose levels for lunch. Correlation between starvation time from breakfast to lunch and iAUC for 2 h after lunch in the low- (**A**), standard- (**B**), and high- (**C**) energy trials. Correlations between starvation time from breakfast to lunch and iAUC 2 h after lunch in the standard (**D**), protein-rich (**E**), fat-rich (**F**), and carbohydrate-rich (**G**) trials.

In the energy trial, no correlation was found between the starvation time from breakfast to lunch (95% CI 4.5–5.4 h, 4.1–5.3 h and 4.4–6.2 h in the low-, standard- and high-energy lunch trial) and the AUC for 4 h after lunch (*P* = 0.949, *P* = 0.895, *P* = 0.637) (Fig. 4A, B, and C). No correlation was found between the starvation time from breakfast to lunch (95% CI 4.6–5.5 h, 4.7–5.9 h, 4.6–5.5 h and 4.4–5.4 h in the standard, protein-rich, fat-rich and carbohydrate-rich trial) and the AUC 4 h after lunch in the balance trial (*P* = 0.770, *P* = 0.449, *P* = 0.953, *P* = 0.191) (Fig. 4D, E, F, and G).

#### Correlation between the difference in starvation time from lunch to dinner and blood glucose levels

**Fig. 5:**
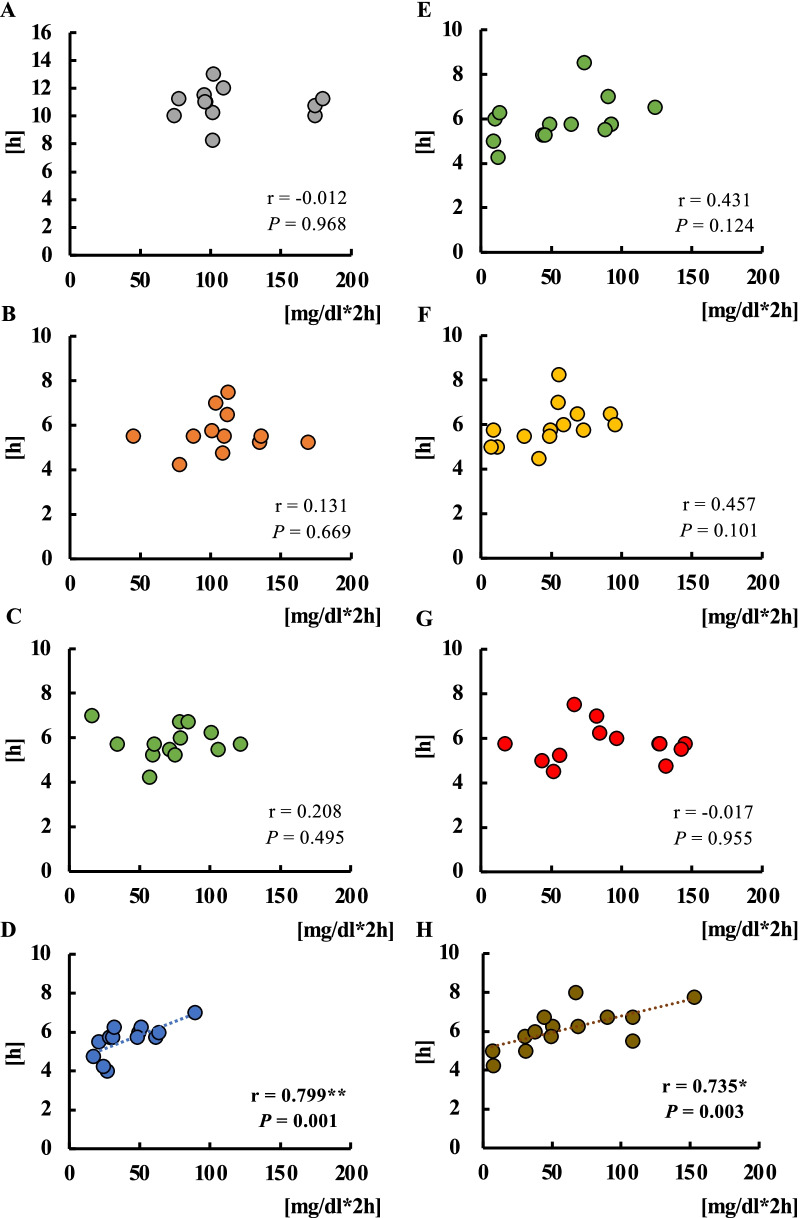
Correlations between starvation time from lunch to dinner and postprandial blood glucose levels for dinner. Correlation between starvation time from lunch to dinner and iAUC for 2 h after lunch in the no (**A**), low- (**B**), standard- (**C**), and high-energy trials (**D**). Correlations between starvation time from lunch to dinner and iAUC 2 h after dinner in the standard (**E**), protein-rich (**F**), fat-rich (**G**), and carbohydrate-rich (**H**) trials.

In the energy trial, on examining the correlation between the starvation time from lunch to dinner (95% CI 10.1–11.5, 5.2–6.2 h, 5.4–6.3 h and 5.1–6.1 h in the no-, low-, standard- and high-energy lunch trial) and the iAUC for 2 h after dinner, a correlation was found only in the high-lunch trial (*P* < 0.01, r = 0.799) (Fig. 5D). In the balance trial, on examining the correlation between the starvation time from lunch to dinner (95% CI 5.3–6.1 h, 5.3–6.4 h, 5.2–6.1 h and 5.5–6.7 h in the standard, protein-rich, fat-rich and carbohydrate-rich trial) and the iAUC for 2 h after dinner, a correlation was found only in the fat-rich trial (*P* < 0.01, r = 0.735) (Fig. 5H).

#### Comparison of the blood glucose levels after dinner on classifying into two groups by mean starvation time in the energy trial

There was a positive correlation between the length of the starvation time from lunch to dinner and blood glucose levels in the high-energy and high-carbohydrate diets (Fig. 5). Therefore, we divided the subjects into two groups: the shorter-starvation group (*n* = 6, starvation time range 4.2–5.6 h) and the longer-starvation group (n = *7*, starvation time range 5.7–7.1 h) by the median mean of starvation time from lunch to dinner in the energy trial.

**Fig. 6:**
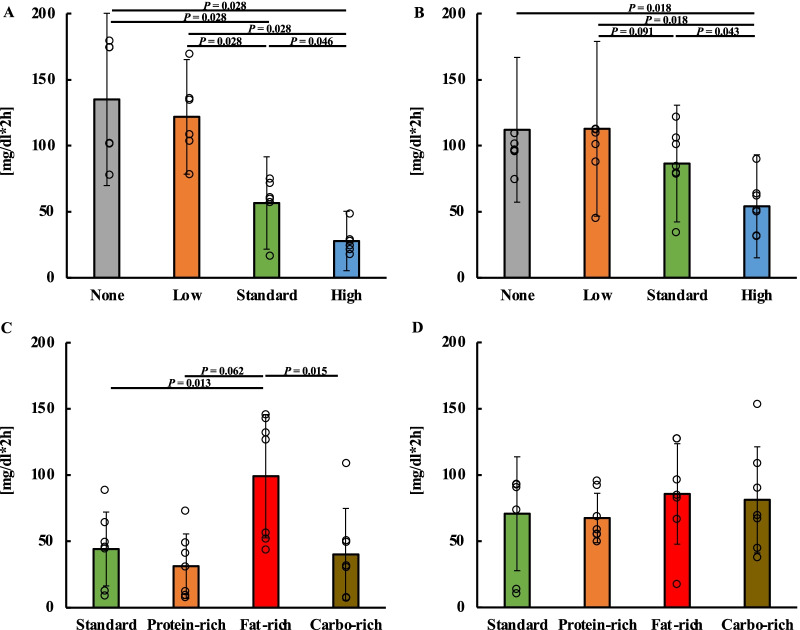
Comparisons of the blood glucose levels after dinner on classifying into two groups by mean starvation time in the energy and balance trials All the data are presented as the mean (standard deviation). iAUC for 2 h after dinner for each trial for the shorter-starvation (**A**) and longer-starvation (**B**) groups in the energy trial. iAUC for 2 h after dinner for each trial for the shorter-starvation (**C**) and longer-starvation (**D**) groups in the balance trial. Fig A and B used the Friedman test. **C** used One-way ANOVA.

In the shorter starvation group, the iAUC for 2 h after dinner was 135.0 (65.2) (95% CI 86.7–183.3) in the no-energy lunch trial, 121.8 (43.4) (95% CI 88.4–155.2) in the low-energy lunch trial, 56.6 (35.0) (95% CI 34.4–78.8) in the standard energy trial and 146.9 (31.1) (95% CI 16.5–39.1) in the high-energy trial. The iAUC for 2 h after dinner was significantly lower in the high-intake trial than that in the no-, low-, and standard-lunch trials (*P* = 0.028, *P* = 0.028, *P* = 0.046 vs high-energy lunch trial). Moreover, it was significantly lower in the standard lunch trial than that in the no and low-energy lunch trials (*P* = 0.028, *P* = 0.028, *P* = 0.046 vs standard-energy trial). In the longer starvation group, the iAUC for 2 h after dinner was 112 (54.8) (95% CI 70.9–153.1) in the no-energy lunch trial, 112.8 (66.1) (95% CI 63.4–162.1) in the low-energy lunch trial, 86.5 (44.2) (95% CI 60.6–112.4) in the standard energy trial, and 54.1 (39.0) (95% CI 35.3–72.9) in the high-energy lunch trial.The iAUC for 2 h after dinner was significantly lower in the high-energy lunch trial than those in the no, low-, or standard-energy lunch trials (*P* = 0.018, *P* = 0.018, *P* = 0.043 vs high-energy lunch trial). Comparing the longer-starvation group with the shorter-starvation group, the standard-energy lunch trial in the shorter starvation group showed lower blood glucose levels after dinner (Fig. 6A, B). We examined the physical characteristics of the shorter- and longer-starvation groups. The starvation times were 5.3 (0.5) and 6.3 (0.1) h in the shorter and longer starvation group. The longer starvation group had significantly longer starvation times (*P* = 0.000) (Additional file 3: Fig. S3A). In addition, the iAUC for 2 h after dinner in the standard trial was 50.0 (23.8) (95% CI 35.0–65.0) and 78.6 (34.5) (95% CI 57.9–99.3) mg/dl*2 h in the shorter and longer starvation group. Comparing the iAUCs for 2 h blood glucose levels after dinner in the standard-lunch trial, the longer-starvation group showed significantly higher values than did the shorter starvation group (*P* = 0.024) (Additional file 3: Fig. S3B). There were no significant differences in BMI or daily energy intake between groups (*P* = 0.587, *P* = 0.723) (Additional file 3: Fig. S3C, D). The step counts were 9786.6 (3786.3) (95% CI 7405.2–12,168.1) and 9653.6 (1289.2) (95% CI 7064.3–12,242.9) steps/day in the shorter and longer starvation group. The MVPA was 99.2 (31.2) (95% CI 79.5–118.8) and 92.4 (37.4) (95% CI 69.7–115.0) min/day in the shorter and longer starvation group. Physical activity levels, such as step counts and MVPA, tended to be higher in the shorter-starvation group than those in the longer-starvation group (*P* = 0.645, *P* = 0.245) (Additional file 3: Fig. S3E, F). The daily intake of dietary fiber was 13.7 (3.0) (95% CI 11.8–15.6) and 13.1 (3.0) (95% CI 11.3–14.9) g in the shorter and longer starvation group. The MEQ score was 64.5 (11.7) (95% CI 57.2–71.9) and 61.6 (7.3) (95% CI 57.2–66.0) in the shorter and longer starvation group. The shorter-starvation group tended to consume more dietary fiber and had higher MEQ scores than the longer-starvation group (*P* = 0.628, *P* = 0.461) (Additional file 3: Fig. S3G, H).

#### Comparison of the blood glucose levels after dinner on classifying into two groups by mean starvation time in the balance trial

We divided the patients into two groups: the shorter starvation group (n = 7, starvation time range 4.4–5.9 h) and the longer starvation group (n = 7, starvation time range 5.9–7.3 h) based on the median mean of starvation time from lunch to dinner in the balance trial.

In the shorter starvation group, the iAUC for 2 h after dinner was 44.3 (27.9) (95% CI 18.5–70.1) in the standard trial, 31.3 (24.4) (95% CI 8.8–53.9) in the protein-rich trial, 99.3 (46.5) (95% CI 56.2–142.3) in the fat-rich trial, and 40.3 (34.7) (95% CI 8.2–72.3) in the carbohydrate-rich trial. The iAUC for 2 h after dinner was significantly higher in the fat-rich trial than those in the standard and carbohydrate-rich trials (*P* = 0.013, *P* = 0.015 vs fat-rich trial) (Fig. 6C). However, in the longer-starvation group, the iAUC for 2 h after dinner was 70.7 (43.0) (95% CI 5.9–186.3) in the standard trial, 67.5 (18.7) (95% CI50.2–84.7) in the protein-rich trial, 85.6 (37.9) (95% CI50.5–120.7) in fat-rich trial and 81.2 (40.0) (95% CI 44.1–118.2) in the carbohydrate-rich trial. There were no significant differences between the trials (*P* = 0.224) (Fig. 6D).

### Relationship between the percentage of energy intake during the usual lunch and postprandial blood glucose levels after dinner during the trial

The energy intake and percentage of energy intake of participants in the energy trial are shown in Table 3A, B. The relationship between the 2 h after dinner iAUC and peak blood glucose level for each trial in the energy trial and the usual percentage of energy intake was examined and found to have no significant correlation (*P* = 0.322, *P* = 0.197 in usual energy intake, *P* = 0.514, *P* = 0.398 in percentage of protein intake, *P* = 0.456, *P* = 0.394 in percentage of fat intake, *P* = 0.325, *P* = 0.321 in percentage of carbohydrate intake) (Additional file 4: Fig. S4).

**Table 3:** Energy intake and percentage of energy intake during daily life for participants

	Protein	Fat	Carbohydrate	kcal	Protein	Fat	Carbohydrate	kcal
	A				B			
Total [g]	100.3(15.2)	86.3(11.1)	329.7(33.7)	2574.2(233.2)	85.1(2.0)	83.1(5.9)	252.2(10.0)	2233.2(112.1)
[%]	16.1	31.1	52.8		16.2	35.7	48.1	
Breakfast [g]	18.0(7.2)	20.2(8.2)	65.8(25.7)	524.5(192.0)	21.5(5.4)	24.6(6.3)	57.0(7.3)	542.2(10.0)
[%]	13.9	35.2	50.9		16.1	41.3	42.6	
Lunch [g]	33.2(8.4)	28.5(5.8)	103.9(15.9)	825.3(135.6)	29.0(3.1)	29.9(3.1)	81.2(7.9)	723.2(62.5)
[%]	16.5	31.8	51.7		16.4	37.9	45.8	
Dinner [g]	45.8(12.5)	36.2(10.2)	139.9(24.1)	1104.7(181.1)	52.5(11.4)	40.0(8.0)	140.8(16.1)	1163.9(189.5)
[%]	17.2	30.5	52.4		18.5	31.8	49.7	
	C				D			
Total [g]	99.6(17.8)	80.9(13.5)	311.4(21.6)	2433.7 ± 77.9	80.7(12.2)	80.7(9.8)	245.3 (31.2)	2052.1 (277.6)
[%]	16.8	30.7	52.5		16.3	16.3	49.5	
Breakfast [g]	23.2(13.4)	19.8(7.7)	67.3(27.2)	539.2 ± 84.1	23.2 (9.2)	23.2(6.4)	67.3(23.3)	589.4(178,7)
[%]	17.1	33.0	49.9		15.9	15.9	46.2	
Lunch [g]	32.2(7.2)	27.8(7.5)	94.3(16.9)	754.3 ± 45.7	26.4 (3.6)	26.4(5.1)	80.4 (11.0)	665.0(91.7)
[%]	17.0	33.1	49.9		16.1	16.1	49.2	
Dinner [g]	40.4(8.1)	30.9(6.9)	130.8(26.3)	1005.0 ± 48.1	29.7(1.9)	29.7 (1.3)	84.6 (4.9)	703.9 (58.2)
[%]	16.8	28.9	54.4		17.4	17.4	49.6	

All data are presented as mean (standard deviation), Energy intake and percentage of energy intake during daily life for men (A) and women (B) participants in the energy trial group. Energy intake and percentage of energy intake during daily life for men (C) and women (D) participants in the balance trial group

The energy intake and percentage of energy intake of the participants in the balance trial are shown in Tables 3C and D. The relationship between the 2 h after dinner iAUC and peak blood glucose level for each trial in the balance trial and the percentage of usual lunch intake was examined. The results showed no significant correlation between the percentage of fat and carbohydrate intake during the usual lunch and postprandial blood glucose levels after dinner. However, there was a positive correlation between the usual percentage of protein intake at lunch and the iAUC at 2 h after dinner (*P* = 0.088, r = 0.473) and a significant positive correlation with the peak blood glucose level at dinner (*P* = 0.041, r = 0.551) (Additional file 5: Fig. S5A, D).

## Discussion

In the first part of the current study, we found that lunch skipping and/or low-energy lunch, such as 200 kcal, caused a higher increase in postprandial tissue glucose at dinner time. It is well known that breakfast skipping provides an increase in postprandial blood glucose levels at lunchtime in comparison with non-skippers [30]. Our present results supplement this result with information on the risk of lunch skipping and/or small lunch food on the high increase in postprandial glucose, or the so-called blood glucose spike. As per the current understanding of the risk of diabetes, small rather than large daily changes in blood glucose levels between minimum and maximum better offer protection against cardiovascular diseases [33]. Therefore, skipping meals may be harmful, but more evidence is needed. In 2015, a nationwide cross-sectional study in Iran (14,286 students aged 7–18) revealed that the frequencies of breakfast, lunch, and dinner skipping were 13.85, 6.8%, and 7.5%, respectively [10]. The National Nutrition Survey in Japan (NNSJ) in 2019 reported that the frequencies of breakfast, lunch, and dinner skipping were 12.1%, 4.0%, and 1.0%, respectively [34]. These data suggest a risk of lunch skipping like that of breakfast skipping. To understand this scenario, we should determine whether breakfast consumption after skipping dinner causes a greater increase in blood glucose levels.

Starvation with time-restricted feeding (TRF) is now a popular way to reduce obesity, risk of cardiovascular disease, abnormalities in lipid metabolism, and diabetes [35, 36]. Men with prediabetes were randomized to TRF (6 h feeding period, with dinner before 3 p.m.) or a control schedule (12 h feeding period) for 5 weeks and were later crossed over to the other schedule. TRF improves insulin sensitivity, β-cell responsiveness, blood pressure, oxidative stress, and appetite [37]. TRF (10 h feeding period) has been reported to have anti-obesity effects in general people and people with obesity, to promote the recovery of abnormal lipid metabolism, and reduce hypertension in people with diabetes [38]. Interestingly, the delayed eating schedule (food intake limit 1200 h–2300 h) for 8 weeks did not cause an anti-obesity (BMI reduction) effect compared to the daytime eating schedule (food intake limit of 0800 h–1900 h) [39]. Thus, the timing and duration of starvation are key factors for the success of anti-obesity treatment. In our crossover intervention experiments, a 10 h fasting time was set after dinner until the next day's breakfast or after breakfast until dinner. Breakfast consumption provided a lower tissue glucose increase than dinner, even though the same meal was consumed at breakfast and dinner [40]. Our recent findings support the risk of daytime starvation on the control of tissue glucose increase and body weight increase, rather than nighttime starvation.

When the combined lunchtime 4 h AUC of glucose and dinner time 4 h AUC of glucose were analyzed, the total glucose AUC was higher in the standard lunch trial than those in the non-, low-, and high-energy lunch trials (Fig. 2E), suggesting that a slightly lower amount of energy than standard lunch (700 kcal) may be required for glucose level control throughout the day.

In the second part of the current experiment, we found that a high-fat lunch provided a higher increase in tissue glucose at dinner than a standard lunch meal, even if participants took the middle lunch size, such as 700 kcal for men and 526 kcal for women. At lunchtime, a high-fat meal provided a significantly lower level of tissue glucose increase compared with a standard PFC-balanced meal. It was reported that the intake of breakfast containing high fat content provided an increase in tissue glucose at lunchtime compared with a standard PFC-balanced breakfast [41], supporting our current result. Although a high-fat meal does not increase tissue glucose levels at lunchtime, attention should be paid to the high increase in tissue glucose levels at dinner time. The combined data of lunch glucose and dinner glucose AUC values were lower than those in the high-carbohydrate trial. The increase in free fatty acids (FFA) in the blood may be increased by high-fat food, and this increased FFA may reduce insulin sensitivity [42].

In the case of high-carbohydrate meals at lunch, glucose levels at lunchtime were strongly increased, but glucose levels at dinner were similar to those observed in the standard lunch trial. On combining lunchtime and dinner time 4 h glucose AUC values, the carbohydrate group showed significantly higher levels than the standard, high protein, and high-fat trials, suggesting that carbohydrate-rich lunches confer a greater risk of high glucose levels throughout the day. In the case of protein-rich meals at lunch, glucose levels tended to reduce the glucose AUC at lunchtime. A review reported that whey proteins, rich in branched-chain amino acids (BCAAs) such as leucine, isoleucine, valine, and lysine may decrease postprandial glucose responses and stimulate insulin secretion in healthy individuals and patients with type 2 diabetes [43, 44]. In addition, protein-rich foods increase GLP-1, and GLP-1 increase may be involved in the second-meal effect [43]. As protein-rich food itself has a weak effect on tissue glucose and protein-rich food provides the second-meal effect, these two effects may be related to low levels of glucose throughout the day.

In this experiment, starvation periods between breakfast and lunch and between lunch and dinner were dependent on the participants’ lifestyle, because we asked them to maintain their mealtime patterns throughout the experiments. Therefore, there were 4–6 h differences between breakfast and lunch, and 4–7 h differences between lunch and dinner. There was no association between the starvation period and standard meal-induced tissue glucose level at lunch in experiments 1 (energy difference of lunch) and 2 (nutrient balance of lunch). The current observation suggests that a balanced and calculated amount of energy meal at breakfast can control the next meal-induced tissue glucose level equally at early or late lunchtime. In future experiments, we aim to investigate whether the composition and/or energy quantity of breakfast has a similar effect on lunchtime. Similarly, we checked whether lunch with different energy quantities or unbalanced PFC percentages affected standard meal-induced tissue glucose levels at early or late dinner time. There was no association between tissue glucose level and starvation period at dinner in the 0 kcal, 200 kcal, and 700 kcal lunch trials, but there was a significant positive correlation between the starvation period and glucose levels in the 1200 kcal trial. There was no association between tissue glucose level and starvation period at dinner in the PFC-balanced, protein-rich, and fat-rich lunch trials; however, there was a significant positive correlation between the starvation period and glucose levels in the carbohydrate-rich lunch trial. Longer starvation with meal skipping augments postprandial glucose increase in the next meal [27–30, 45]. Although the mechanism of these results is currently unknown, insulin secretion and/or sensitivity [28, 30], GLP-1 secretion [44], and other unknown factors may be involved in this second-meal effect.

As starvation between lunch and dinner may affect glucose levels at dinner, we divided the participants into two groups (shorter starvation group and longer starvation group) by the median value of the starvation period. The shorter starvation group showed lower tissue glucose levels at dinner in the standard lunch trial compared with the longer starvation group. In general, later dinners are known to produce higher glucose levels [25]; therefore, higher levels of glucose at dinner were observed in the longer-starvation group in the standard and high-energy lunch trials. In the next experiment, we examined the characteristics of the two shorter and longer groups. The shorter starvation group showed higher physical activity [21], higher dietary fiber intake [19, 20], and a tendency towards morningness in the MEQ score [21]. These tendencies are related to a reduction in blood glucose levels. The shorter starvation group provided higher tissue glucose levels at dinner time in the high-fat lunch trial than the longer starvation group. Furthermore, FFAs block insulin action [42, 46]. FFA production by high-fat food may be high at an early dinner time and may interfere with the insulin effect, but may not maintain high levels at a later dinner time.

In the current experiment, we examined whether the intervention of energy and PFC balance differences at lunch affected tissue glucose levels at dinner time. Therefore, the daily intake of lunch energy and PFC balance assessed by FFQ may be related to lunch intervention on tissue glucose levels at dinner. There was no association between daily lunch energy (kcal), lunch PFC balance (%), and glucose levels at dinner in the standard-lunch trial. Further, we investigated whether the daily lunch protein, fat, and carbohydrate ratio (%) affect glucose levels at dinner time in the protein-rich, fat-rich, and carbohydrate-rich lunch trials, respectively. There was a strong positive association between the daily protein ratio (%) and maximum values (*P* = 0.041, r = 0.551) and 2-h iAUC (*P* = 0.088, r = 0.473) of tissue glucose levels at dinner. As protein-rich lunches led to low levels of tissue glucose at dinner due to the second meal effect, we hypothesized a negative association between daily lunch protein quantity and tissue glucose levels at dinner. Although the possible mechanism of the present results is unclear, the daily intake of protein-rich lunches may cause the downregulation of mechanisms of the second-meal effect.

Physical activity may affect the control of tissue glucose changes, and we examined the association between standard dinner-induced glucose increase and physical activity. There was no association between these two factors, suggesting that physical activity levels and tissue glucose changes did not influence the present study.

## Limitation of experiments

Our study has some limitations. First, the participants were healthy young adults. Our results may not be applicable to other subjects, such as healthy older men and women, or patients with diabetes. However, only a few studies have focused on lunch. Therefore, the results of this study are important as they may lead to future research. Second, the effects of differences in dietary intake on non-trial days on blood glucose fluctuations cannot be ruled out. A test meal was served on each trial day. However, the meals on other days were not controlled and may have influenced blood glucose fluctuations. On the other hand, the participants were instructed to maintain their normal lifestyle throughout the experiment. Therefore, the effect of differences in food intake on non-trial days on blood glucose levels is considered weak. Prospects for future research include study protocols for controlling diet during the entire study period with less influence of the usual diet, which may lead to more detailed clarification.

## Supplementary Information

**Additional file 1**. **Fig. S1**: Relationship between physical activity levels during each trial period and iAUC for 2 h after dinner. Correlation between physical activity levels during the energy trial period and iAUC for 2 h after dinner in the standard trial (A, B). Correlation between physical activity levels during the balance trial period and iAUC for 2 h after dinner in the standard trial (C and D). MVPA: moderate-to-vigorous physical activity.

**Additional file 2**. **Fig. S2**: Glucose excursion for 24 h in the energy trial group (A), and the balance trial group (B). Significant differences between trials after lunch at each 15-minute time interval in the energy trial group. *P < 0.05, **P < 0.01 (between no- and low-energy lunch trial), †P < 0.05, ††P < 0.01, †††P < 0.001 (between no- and standard-energy lunch trial), ‡P < 0.05,‡‡P < 0.01, ‡‡‡P < 0.001 (between no- and high-energy lunch trial), &P < 0.05, &&P < 0.01, &&&P < 0.001 (between low- and standard-energy lunch trial), $P < 0.05, $$P < 0.01, $$$P < 0.001 (between low- and high-energy lunch trial), ##P < 0.01, ###P < 0.001 (between standard- and high-energy lunch trial) (Two-way ANOVA) (C). Significant differences between trials after dinner at each 15-minute time interval in the energy trial group. ††P < 0.01, †††P < 0.001 (between standard and fat-rich trial), ‡P < 0.05 (between standard and carbohydrate-rich trial), $P < 0.05, $$P < 0.005, $$$P < 0.001 (between protein-rich and carbohydrate-rich trial), #P < 0.05 (between fat-rich and carbohydrate-rich trial) (D). Significant differences between trials after lunch at each 15-minute time interval in the balance trial group. †P < 0.05 (between no- and standard-energy lunch trial), ‡P < 0.05, ‡‡P < 0.01, ‡‡‡P < 0.001 (between no- and high-energy lunch trial), $P < 0.05, $$P < 0.01, $$$P < 0.001 (between low- and high-energy lunch trial), #P < 0.01, ##P < 0.005, ###P < 0.001 (between standard- and high-energy lunch trial) (Two-way ANOVA) (E). Significant differences between trials at each 15-minute time interval in the balance trial group †P < 0.05 (between standard and fat-rich trial), $P < 0.05, $$P < 0.01 (between low- and high-energy lunch trial) (Two-way ANOVA) (F).

**Additional file 3**. **Fig. S3**: Characteristics after classifying into two groups by mean starvation time. Starvation time (A), iAUC for 2 h after dinner (B), body mass index (C), energy intake (D), step counts (E), moderate-to-vigorous physical activity (F), intake of dietary fiber (G), and MEQ (H). MEQ; Morningness–Eveningness Questionnaire. A t-test was used to test for differences between groups.

**Additional file 4**. **Fig. S4**: Association between lunch energy intake during daily life and after-dinner blood glucose levels in the energy trial. Relationship between intake (A), percentage of protein (B), percentage of fat (C), percentage of carbohydrate (D) during daily life for lunch, and iAUC for 2 h after dinner in the standard trial. Relationship between intake (E), percentage of protein (F), percentage of fat (G), percentage of carbohydrates (H) during daily life for lunch, and peak blood glucose levels after dinner in the standard trial.

**Additional file 5**. **Fig. S5**: Association between lunch energy intake during daily life and after-dinner blood glucose levels in the balance trial. Relationship between percentage of protein during daily life for lunch and iAUC for 2 h after dinner in the protein-rich trial (A). Relationship between the percentage of fat during daily life for lunch and iAUC for 2 h after dinner in the fat-rich trial (B). Relationship between percentage of carbohydrates during daily life for lunch and iAUC for 2 h after dinner in the carbohydrate-rich trial (C). Relationship between percentage of protein (D), fat (E), and carbohydrate (F) during daily life for lunch and peak blood glucose levels after dinner in the standard trial.

## Data Availability

All data generated or analyzed during this study are included in this published article and its additional files.

## Abbreviations

AUC: Area under the curve
BCAAs: Branched-chain amino acids
BIA: Bioelectrical impedance analysis
BMI: Body mass index
CGM: Continuous glucose monitoring
FFA: Free fatty acids
FFQ: Food frequency questionnaire
HPI: Healthy purchase index
iAUC: Incremental area under the curve
MEQ: Morningness–eveningness questionnaire
MVPA: Moderate-to-vigorous physical activity
NNSJ: National nutrition survey in Japan
PFC Balance: Protein, fat, and carbohydrate balance
TRF: Time-restricted feeding
