# Supplementary figures and images for: Effect of lunch with different calorie and nutrient balances on dinner-induced postprandial glucose variability
Source: Nutr Metab (Lond). 2022 Sep 24;19:65. doi: 10.1186/s12986-022-00704-1 (PMC9509610; doi:10.1186/s12986-022-00704-1)

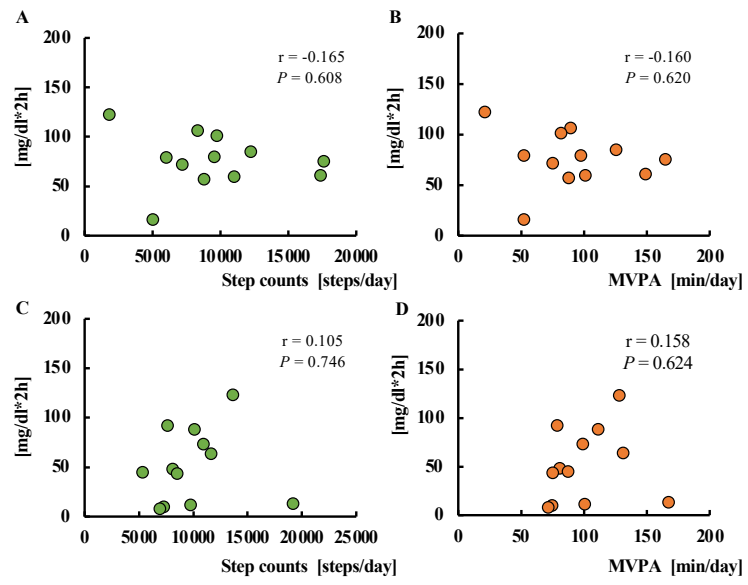

Supplement: Supplementary file 1 — Additional file 1. Fig. S1: Relationship between physical activity levels during each trial period and iAUC for 2 h after dinner. Correlation between physical activity levels during the energy trial period and iAUC for 2 h after dinner in the standard trial (A, B). Correlation between physical activity levels during the balance trial period and iAUC for 2 h after dinner in the standard trial (C and D). MVPA: moderate-to-vigorous physical activity. [file 12986_2022_704_MOESM1_ESM.pdf]

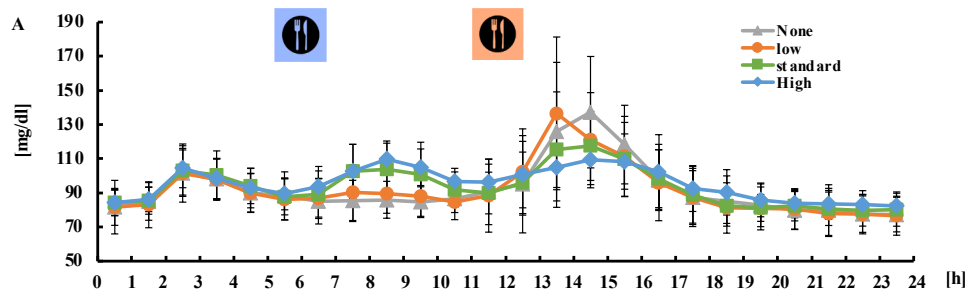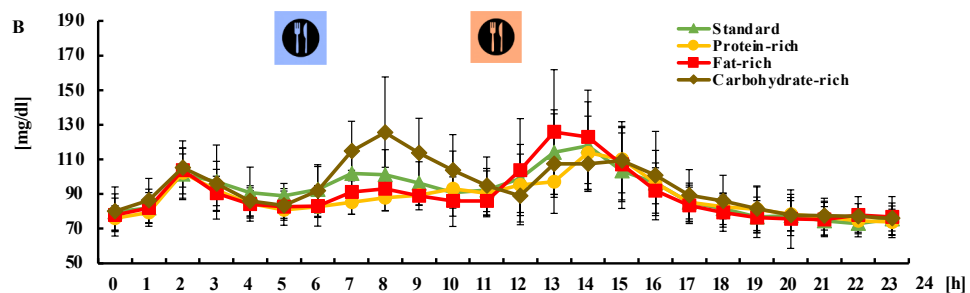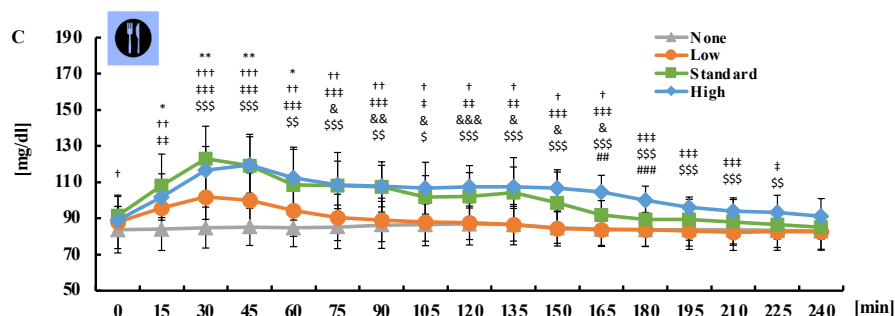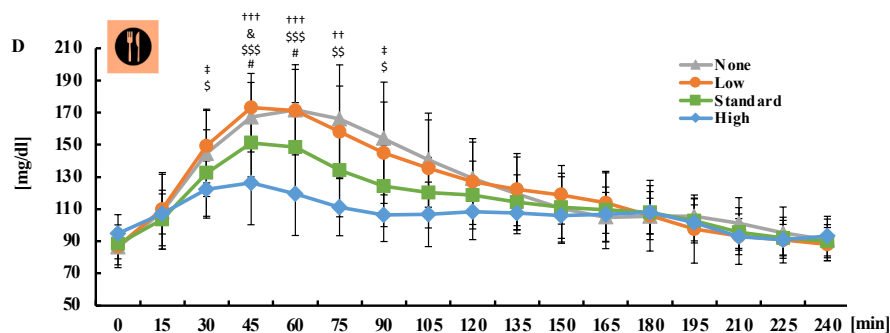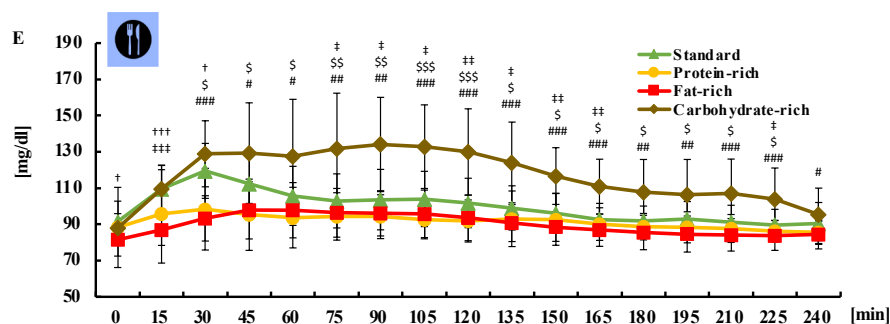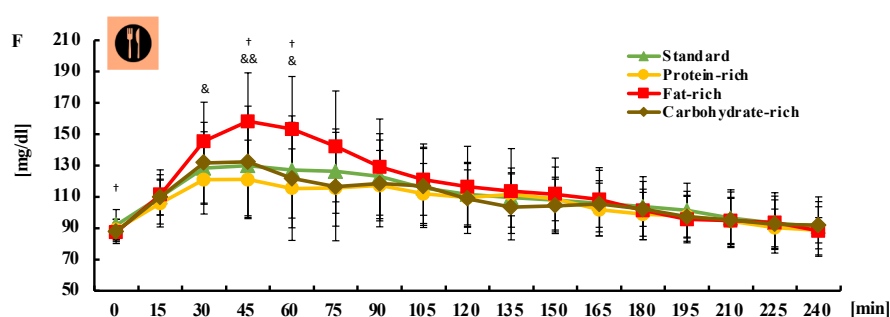

Supplement: Supplementary file 2 — Additional file 2. Fig. S2: Glucose excursion for 24 h in the energy trial group (A), and the balance trial group (B). Significant differences between trials after lunch at each 15-minute time interval in the energy trial group. *P < 0.05, **P < 0.01 (between no- and low-energy lunch trial), †P < 0.05, ††P < 0.01, †††P < 0.001 (between no- and standard-energy lunch trial), ‡P < 0.05,‡‡P < 0.01, ‡‡‡P < 0.001 (between no- and high-energy lunch trial), &P < 0.05, &&P < 0.01, &&&P < 0.001 (between low- and standard-energy lunch trial), $P < 0.05, $$P < 0.01, $$$P < 0.001 (between low- and high-energy lunch trial), ##P < 0.01, ###P < 0.001 (between standard- and high-energy lunch trial) (Two-way ANOVA) (C). Significant differences between trials after dinner at each 15-minute time interval in the energy trial group. ††P < 0.01, †††P < 0.001 (between standard and fat-rich trial), ‡P < 0.05 (between standard and carbohydrate-rich trial), $P < 0.05, $$P < 0.005, $$$P < 0.001 (between protein-rich and carbohydrate-rich trial), #P < 0.05 (between fat-rich and carbohydrate-rich trial) (D). Significant differences between trials after lunch at each 15-minute time interval in the balance trial group. †P < 0.05 (between no- and standard-energy lunch trial), ‡P < 0.05, ‡‡P < 0.01, ‡‡‡P < 0.001 (between no- and high-energy lunch trial), $P < 0.05, $$P < 0.01, $$$P < 0.001 (between low- and high-energy lunch trial), #P < 0.01, ##P < 0.005, ###P < 0.001 (between standard- and high-energy lunch trial) (Two-way ANOVA) (E). Significant differences between trials at each 15-minute time interval in the balance trial group †P < 0.05 (between standard and fat-rich trial), $P < 0.05, $$P < 0.01 (between low- and high-energy lunch trial) (Two-way ANOVA) (F). [file 12986_2022_704_MOESM2_ESM.pdf]

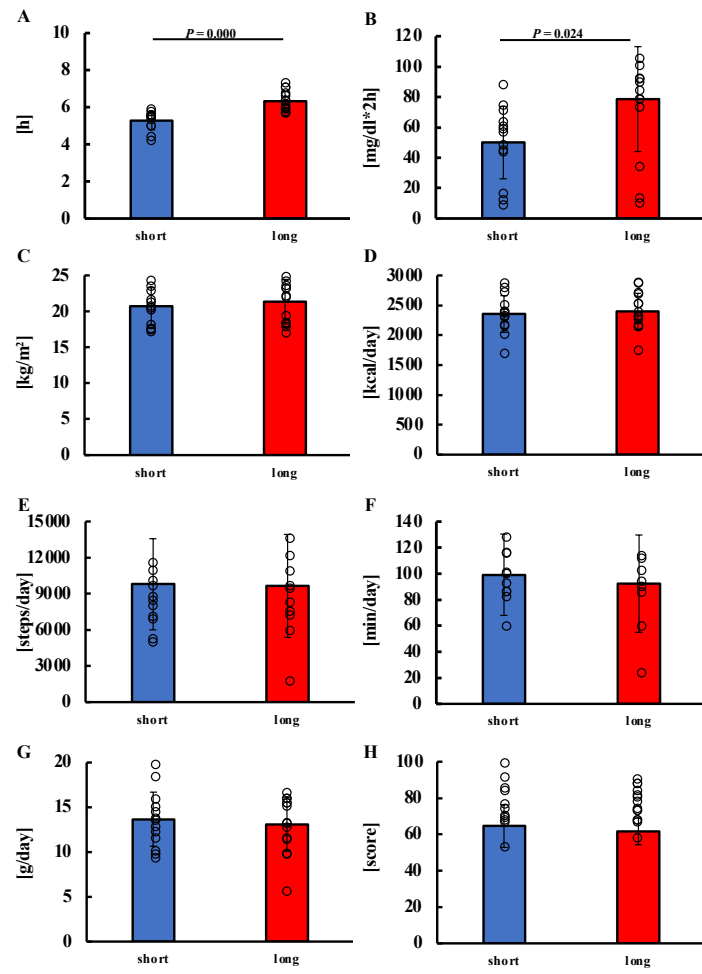

Supplement: Supplementary file 3 — Additional file 3. Fig. S3: Characteristics after classifying into two groups by mean starvation time. Starvation time (A), iAUC for 2 h after dinner (B), body mass index (C), energy intake (D), step counts (E), moderate-to-vigorous physical activity (F), intake of dietary fiber (G), and MEQ (H). MEQ; Morningness–Eveningness Questionnaire. A t-test was used to test for differences between groups. [file 12986_2022_704_MOESM3_ESM.pdf]

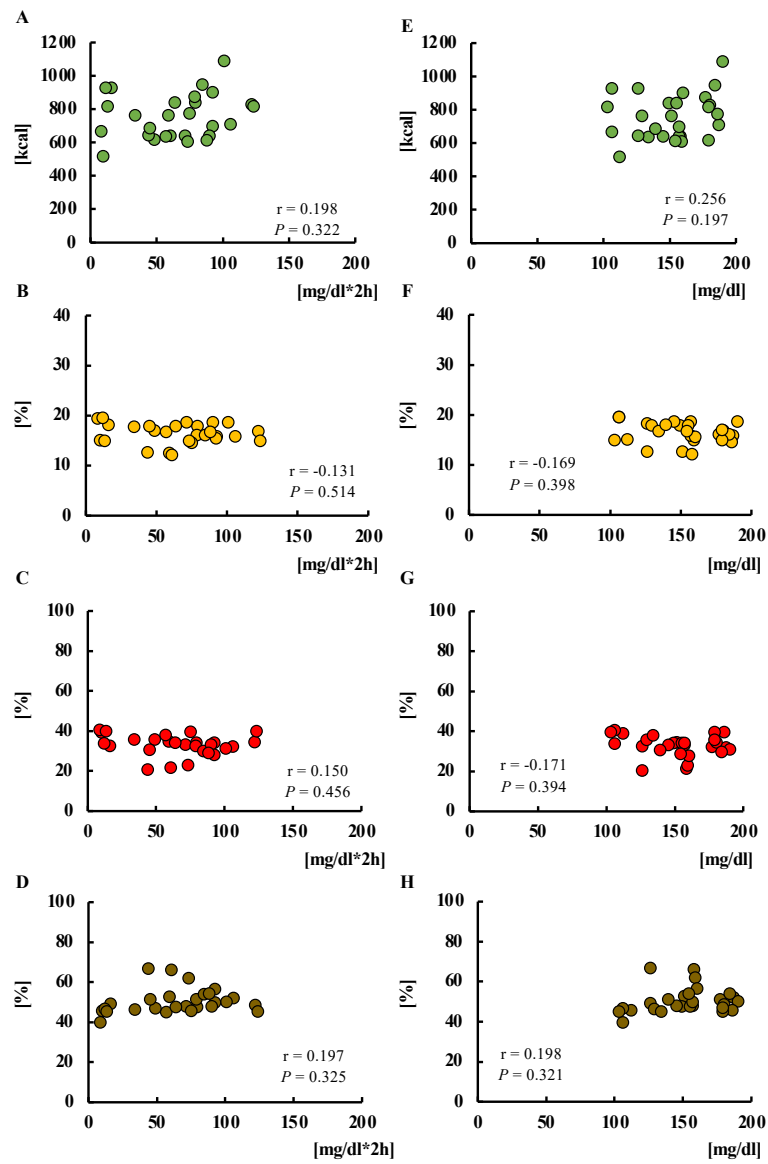

Supplement: Supplementary file 4 — Additional file 4. Fig. S4: Association between lunch energy intake during daily life and after-dinner blood glucose levels in the energy trial. Relationship between intake (A), percentage of protein (B), percentage of fat (C), percentage of carbohydrate (D) during daily life for lunch, and iAUC for 2 h after dinner in the standard trial. Relationship between intake (E), percentage of protein (F), percentage of fat (G), percentage of carbohydrates (H) during daily life for lunch, and peak blood glucose levels after dinner in the standard trial. [file 12986_2022_704_MOESM4_ESM.pdf]

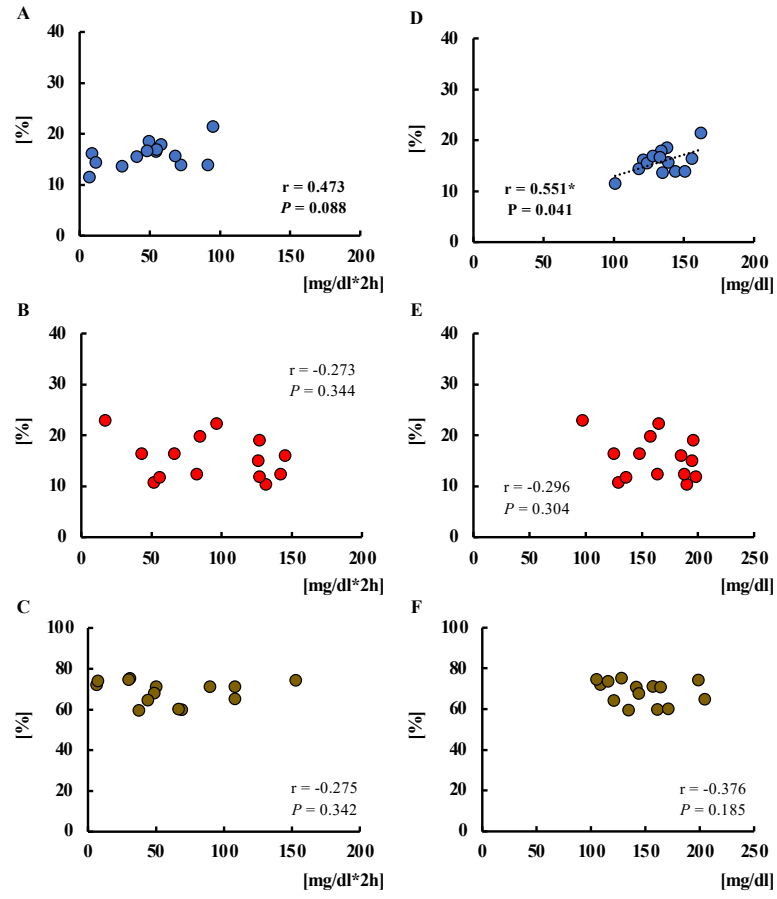

Supplement: Supplementary file 5 — Additional file 5. Fig. S5: Association between lunch energy intake during daily life and after-dinner blood glucose levels in the balance trial. Relationship between percentage of protein during daily life for lunch and iAUC for 2 h after dinner in the protein-rich trial (A). Relationship between the percentage of fat during daily life for lunch and iAUC for 2 h after dinner in the fat-rich trial (B). Relationship between percentage of carbohydrates during daily life for lunch and iAUC for 2 h after dinner in the carbohydrate-rich trial (C). Relationship between percentage of protein (D), fat (E), and carbohydrate (F) during daily life for lunch and peak blood glucose levels after dinner in the standard trial. [file 12986_2022_704_MOESM5_ESM.pdf]
